# Recurring flood distribution patterns related to short-term Holocene climatic variability

**DOI:** 10.1038/srep16398

**Published:** 2015-11-09

**Authors:** Gerardo Benito, Mark G. Macklin, Andrei Panin, Sandro Rossato, Alessandro Fontana, Anna F. Jones, Maria J. Machado, Ekaterina Matlakhova, Paolo Mozzi, Christoph Zielhofer

**Affiliations:** 1 Museo Nacional de Ciencias Naturales, CSIC, Serrano 115 bis, 28006 Madrid, Spain; 2 Centre for Catchment and Coastal Research and the River Basin Dynamics and Hydrology Research Group, Department of Geography and Earth Sciences, Aberystwyth University, Ceredigion SY23 3DB, UK; 3 Innovative River Solutions, Institute of Agriculture and Environment, Massey University, Private Bag 11 222, Palmerston North, 4442, New Zealand; 4 Lomonosov Moscow State University, Faculty of Geography, Lengory 1, Moscow, 119991, Russia; 5 University of Padua, Department of Geosciences, Via Gradenigo, 6. 35131 Padova, Italy; 6 School of Geography, Planning and Environmental Policy, University College Dublin, Newman Building, Belfield, Dublin 4, Ireland; 7 Leipzig University, Institute of Geography, Johannisallee 19a, 04103 Leipzig, Germany

## Abstract

Millennial- and multi-centennial scale climate variability during the Holocene has been well documented, but its impact on the distribution and timing of extreme river floods has yet to be established. Here we present a meta-analysis of more than 2000 radiometrically dated flood units to reconstruct centennial-scale Holocene flood episodes in Europe and North Africa. Our data analysis shows a general increase in flood frequency after 5000 cal. yr BP consistent with a weakening in zonal circulation over the second half of the Holocene, and with an increase in winter insolation. Multi-centennial length phases of flooding in UK and central Europe correspond with periods of minimum solar irradiance, with a clear trend of increasing flood frequency over the last 1000 years. Western Mediterranean regions show synchrony of flood episodes associated with negative phases of the North Atlantic Oscillation that are out-of-phase with those evident within the eastern Mediterranean. This long-term flood record reveals complex but geographically highly interconnected climate-flood relationships, and provides a new framework to understand likely future spatial changes of flood frequency.

Recent climate change has raised awareness about potential alterations in flooding and flood hazards. Detection of trends in extreme floods at river gauging stations is difficult due to the often geographically sparse and short records, even where catchments and drainage networks are not affected by land-use change and river regulation. Fluvial geomorphological and sedimentary records contain evidence of past hydrological events, which can be linked to Earth’s climate variability. Chronological control of Holocene and Late Pleistocene fluvial sedimentary archives has improved greatly in recent decades, and has facilitated their use as records of extreme hydrological events in catchments worldwide^1^,^2^. Although we cannot assume that these long-term record floods and flood patterns will be reproduced in the future, they do hold key knowledge to understand the effect of multi-decadal climate variability on extreme flooding at regional and global scales.

A major achievement in the last decade has been the development of meta-analysis for large databases of ^14^C-dated Holocene flood units^3^. This approach facilitates comparison of probability-based flood series with climate and human impact proxy records, to better constrain the factors that control extreme events, and to inform present and future flood-risk assessment. Here we present a reconstruction of Holocene flooding events based on the meta-analysis of more than 2000 ^14^C and OSL dated flood units from twelve regions in Europe and North Africa, selected on the basis of availability of high quality chronological data on extreme fluvial events (Fig. 1). The dated fluvial deposits comprise slackwater and boulder berm flood sediments representing individual palaeoflood events, as well as flood units from alluvial floodplain and flood basin environments that record, in some instances, single large floods and changes in the discharge-sediment load over multi-decadal periods. For the first time, this reveals spatially coherent flood distribution patterns, which are repeated through time, with synchronous (in-phase) and asynchronous (out-of-phase) episodes of hydrological activity in response to shifts in atmospheric circulation modes associated with Holocene climate variability.

The number of flood episodes varies between regions with four recorded in northeast Morocco, ten in Tunisia and the East European Plain, and sixteen in the UK (Supplementary Table S1). The length of flooding episodes also varies between ~100 years, for the most recent period, to 700 years in the early Holocene where the number of dated samples is small and the errors in dating and calibration process are larger. The analysis indicates that several flooding episodes were synchronous in six of the study areas (highlighted in Supplementary Table S1). Since the duration of these overlapping flooding phases varies between regions, multi-centennial flood episodes were divided into 100-yr bins. A cross-correlation analysis shows the number of 100-yr intervals where there is an overlap of flooding episodes between regions (Supplementary Table S2). The percentage of overlapping 100-yr bins in each region indicates the degree of record synchrony and regions having the highest percentage are characterised by similar hydroclimatic conditions. The west Iberian Peninsula shows a high number of overlapping centennial and multi-centennial length flooding periods with east Iberia, southern France and Tunisia, and the lowest correlation with the eastern Mediterranean and East European Plain. Other regional correlations are: (i) UK with Germany, northeast Italy, and southern France; (ii) East European Plain with Germany, southern France and southern Italy; (iii) Tunisia with northeast Morocco and Iberian Peninsula; and (iv) eastern Mediterranean with East European Plain and Tunisia.

To assess climatic drivers of centennial and multi-centennial flood variability, the aggregate record has been divided into Mediterranean regions and northwest, central and eastern Europe (Fig. 2). In the Mediterranean sector, the most widespread period of flooding occurred between 7500–7000 and at 2500–2000, with other minor phases centred at 4600, 3900, 3000, 1500, 900 and ca 300 cal. BP (Fig. 3d,e). There was also a marked decrease (almost no records) in major floods during the period 6500–5000 cal. BP, followed by an increase in fluvial activity over the last 5000 years. In northwest and northern Europe, there is less synchrony of flood periods between regions than in the Mediterranean, suggesting a lower sensitivity to extreme hydroclimatic changes (i.e. lower hydrological variance; Fig. 3d,e).

## Discussion

**Figure 2:**
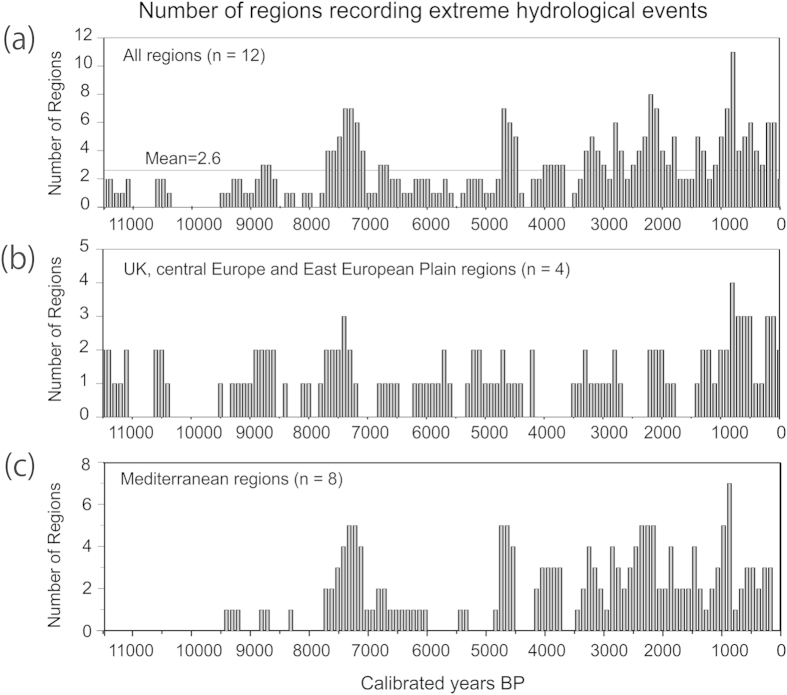
Temporal distribution (100-yr bin intervals) of the number of regions showing synchronised flood activity. (**a**) All regions included in this study (n = 12). (**b**) Regions within the northern temperate climatic zone (UK, Germany, Poland and East European Plain). (**c**) Regions of the conterminous Mediterranean (western and eastern Spain, Southern France, north-eastern Italy, southern Italy, south-eastern Mediterranean, Tunisia and north-east Morocco).

**Figure 3:**
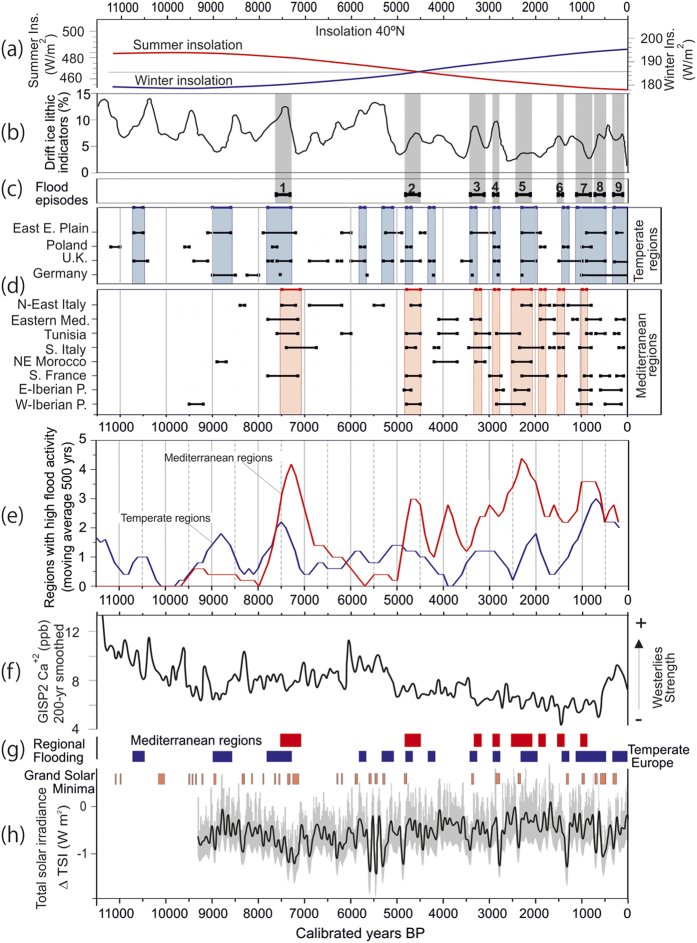
(**a**) Summer and winter insolation curves from 40°N^28^ showing a decrease in summer insolation and an increase in winter insolation since their maximum in the early Holocene around 10 ka. An inflexion of both curves occur ca. 5000 cal. BP. We assume that increase and/or decrease in insolation affects seasonal temperature at a regional scale; (**b**) North Atlantic drift ice index^8^; (**c**) Extreme flood episodes (FE: 1–9) based on the fluvial record from 12 regions (see Supplementary Table S1 for more details); (**d**) Horizontal bars showing age ranges of episodes of increased flooding compiled for this paper based on HEX project groups and previously published studies. Vertical shaded bars show main flood periods for European temperate climatic regions (blue) and Mediterranean regions (red); (**e**) Temporal distribution of increasing river flooding over the Mediterranean and Temperate climatic regions based on the number of regions showing flood activity. Moving average (500 yrs) based on data at 100-yr intervals. Note a change in flood activity pattern in the Mediterranean region after 5 ka; (**f**) Gaussian smoothed (200 yr) GISP2 calcium (nss. Ca^++^) ion proxy for the strength of the westerlies^29^ (zonal flow); (**g**) Flood episodes recorded in temperate and Mediterranean regions (as in d); (**h**) Reconstructed TSI anomalies (100-year lowpass filtered; grey shading: one standard deviation uncertainty range) for the past 9300 years^30^. The reconstruction is based on ^10^Be and calibrated using the relationship between instrumental data of the open magnetic field, which modulates the production of ^10^Be, and TSI for the past four solar minima. Anomalies are relative to the 1976–2006 mean value (1366.14 Wm^–2^). The vertical orange bars correspond to the grand minima in solar activity or periods with very low sunspot number during at least two consecutive decades^31^.

A major issue of current debate in climate change impact science is the effect of climate variability on the spatial-temporal distribution of extreme hydrological events and their synchrony at regional, continental and global scales. Before 5000 cal. BP the synchrony of flood periods across Europe and North Africa was low with only one period occurring in multiple regions (ca. 7500–7200 cal. BP). The first flooding period recognized in the Mediterranean region (7500–7000 cal. BP) occurred during the second part of the sapropel 1 that has been correlated with higher discharges of fresh waters in the eastern Mediterranean Sea^4^. After 5000 cal. BP the aggregated fluvial record shows higher variability and a greater number of widespread flooding episodes (Figs 2 and 3). Other records of flooding from European lakes also show an increase in flooding and storminess after 5000 cal. BP^5^, indicating a climatically-controlled change in flood occurrence and magnitude. In Mediterranean lakes two major phases of palaeohydrological change at ca. 8000–7000 cal. BP and 4500–4000 cal. BP have been documented^6^ which overlap with episodes of increased flooding at 7600–7300 and ~4000 cal. BP. These palaeohydrological changes were attributed to two major forcing mechanisms: orbitally-driven insolation and deglaciation. The increase of orbitally-driven winter insolation during the Holocene (Fig. 3a) has affected seasonal temperatures, although several studies have highlighted the nonlinearity and spatial heterogeneity in regional temperature response^7^. As winter insolation in the Northern Hemisphere increased after 5000 cal. BP the heat exchange with high latitudes favoured a decrease in the strength of the westerlies leading to wetter and milder winters. In Mediterranean regions, a greater number of flood episodes during the late Holocene suggest more frequent meridional flow enhancing cyclogenesis.

Flood episodes in Europe show a good agreement with Holocene ice-rafted debris (IRD) events in the North Atlantic Ocean^8^ associated with the cooling of the ocean surface (Fig. 3b). In the Atlantic-Eurasian region episodes of ice-rafting correspond with phases of dominant meridional circulation with a shift of the main atmospheric streams toward lower latitudes that generate slow-moving cyclonic perturbations and rain^9^. Meridional atmospheric circulation is related to negative mode of the North Atlantic Oscillation (NAO) and Arctic Oscillation (AO) indexes^10^, commonly associated with severe flooding in south-west Europe^11^. Similarly, periods of increased flooding during the Holocene matching with cooling of the North Atlantic would indicate a negative NAO-like circulation. In the East-European Plain (data mostly from central Russia) six out of ten flood episodes occurred during periods of higher IRD fluxes, and three corresponded to transition periods to warmer conditions (Fig. 3b). Regions within the central Mediterranean (Tunisia, Morocco, southern and northern Italy) showed a mixed response to cooling in the North Atlantic region, with geographically variable flood response to IRD-related cooling periods at 1400, 2800 and 4200 cal. BP.

Holocene climate variability is strongly controlled by the dynamics of atmospheric circulation^10^ and, as a result, the spatial-temporal distribution of floods has changed with time (Fig. 3f,g). At the continental scale, atmospheric circulation patterns are strongly influenced by semi-permanent features of the atmospheric pressure fields in the North Atlantic (Icelandic Low/Azores High), and in Eurasia (Siberian High -SH- and Scandinavian high/low pressure). The SH influences weather in the eastern Mediterranean and Europe as far west as Italy, and the Scandinavian pressure field affects patterns of severe weather in Central Europe and in the UK. For example, Holocene flood histories in the western and eastern Mediterranean show opposite multi-decadal flood patterns (Supplementary Tables S1 and S2) with a similar see-saw pattern also recognised in lake records during the Medieval Climatic Anomaly^12^. This flood pattern asymmetry across the Mediterranean indicates that some atmospheric circulation patterns associated with Holocene climatic anomalies have persisted over multi-centennial time scales.

**Figure 4:**
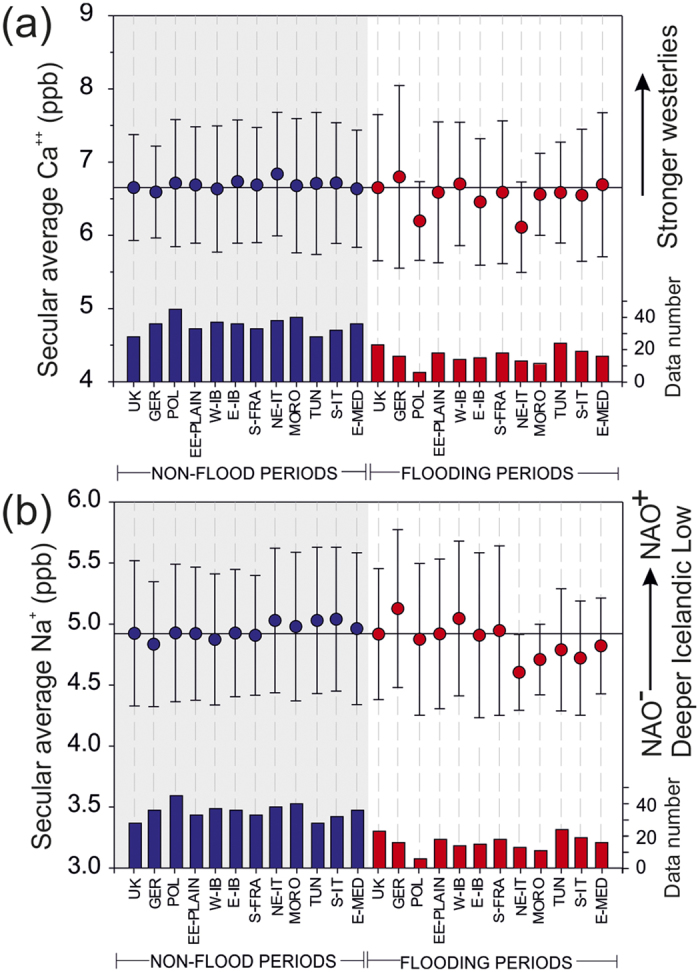
Average values and one standard deviation of nss Ca^++^ and ss Na^+^ deposited in the ice core GISP2^29^ calculated for 100-yr bin intervals of flood activity periods (red dots) and those for periods with a lack of recorded flood activity (blue dots). (**a**) Averaged secular nss Ca^++^ shows lower values during flood activity periods in the Mediterranean regions indicating a positive correlation with weaker westerlies (September-October-November). (**b**) Averaged secular content of ss Na^+^ decreased during the flood activity periods of central Mediterranean regions being interpreted as increases in surface pressure over the Icelandic Low and predominant negative NAO mode during December–January–February. Interpretation of GISP2 aerosols as proxies for atmospheric circulation was taken from previous work^10^.

Recent studies suggest that the mean atmospheric state of the early Holocene (ca. 11,700–8000 cal. BP) resembled the positive phase of the Arctic/North Atlantic Oscillation^13^. Our Holocene fluvial record would support this view and indicates that a dominant westerly (zonal) flow pattern during the early Holocene led to reduced flooding, particularly in the Mediterranean regions. A progressive increase of flood episodes started ca. 5000 cal. BP suggesting a major shift in atmospheric circulation (higher frequency of negative-like NAO; Fig. 3d). In general, periods of higher flood frequency (e.g. 7500–7100 and 2400–2100 cal. BP) coincide with low values of non-sea-salt calcium (nss Ca^++^) dust concentration in the GISP2 ice core (Figs 3f and 4a), which is interpreted to reflect a weakening of high-latitude westerlies^10^,^14^. Conversely peaks of nss Ca^++^ are associated with reduced flood activity (e.g. 6000–5000 cal. BP; ca. 8500–8200 cal. BP). A detailed analysis of secular average content of GISP2 aerosols associated with 100-yr interval of regional flood vs non-recorded flood activity shows significant decrease in contents of sea salt sodium (ss Na^+^) during flood activity periods particularly in the Mediterranean regions (Fig. 4b). Low concentrations of ss Na^+^ have been related to a southwards displacement of the jet stream and storm tracks (negative NAO-like circulation) towards the Mediterranean^10^.

Changes in the spectral solar irradiance, in particular the ultraviolet (UV) spectrum, can influence the tropospheric circulation through dynamical coupling, affecting the North Atlantic circulation in the Eurasian region^15^. Several studies have related periods of a long-term reduction in solar radiation (e.g. Homeric minimum, ca. 2750 cal. BP) with rapid and widespread cooling and relatively wet conditions in the North Atlantic European region^16^. Most episodes of flooding in northwest and northern Europe region match with multi-decadal periods of grand solar minima (Fig. 3g,h). However, in the Mediterranean regions anomalous periods of high flood frequency occurred mainly during periods of increased solar activity. Therefore, the sensitivity of flood occurrence to cyclical variations in solar forcing is higher in northern Europe, whereas in the Mediterranean region it is unlikely to be a major forcing mechanism.

The Holocene flood patterns presented here reveal complex, highly spatially interconnected climate-flood relationships resulting in a multi-decadal to secular variability in flood frequency with strong in-phase or out-of-phase relations between regions. Our analysis suggests that anthropogenic climate change will result in significant spatial variability in flooding both in Europe and North Africa. The main temporal trends are increasing flooding in northern and central Europe and decreasing frequency in western Mediterranean regions during the last centuries. Our palaeoflood evidence demonstrates non-stationarity of flood frequency at multi-decadal time scale is more marked in the Mediterranean than in Northwest and Northern Europe. Regional changes in flood extremes are associated with switches in predominant NAO phase, indicating their high potential to model (as covariates) climate-related impacts on flood risk assessment.

## Materials and Methods

The database of dated river floods comprises: (1) western and eastern Iberian Peninsula (123 ^14^C dates^11^), (2) southern France (38 ^14^C and 6 OSL dates), (3) north-east Morocco (30 ^14^C dates), (4) eastern Mediterranean (47 OSL and 31 ^14^C dates); (5) catchments in north-east Italy draining the southern rim of the eastern Alps (136 ^14^C dates^17^), and (6) the East European Plain in the central part of Russia (553 ^14^C and 10 OSL dates^18^). These new regional databases extend and complement the analysis of previous studies undertaken elsewhere in Europe based on ^14^C dates from fluvial sediments related to floods and enhanced fluvial activity episodes within the UK (252 dates^19^,^20^), Poland (331 dates^21^), Spain (74 dates^22^), Southern Italy (34 dates^23^), Tunisia (103 dates^24^), and Germany (148 dates^25^), which are included in this new analysis, making a total of 2038 radiometrically dated flood units.

The regional databases were constructed based on unified criteria^19^ regarding the sedimentary environments and the selection of dates associated with a change in sedimentation rate indicative of flood activity. The meta-data record for each dated sample includes its location, site characteristics (drainage area, altitude), type of organic material, stratigraphic position, and whether the fluvial unit from which the age dating was obtained represents a period of river channel/floodplain activity or stability^24^. Each individual ^14^C and OSL date and its stratigraphic setting was analysed to determine whether it constituted a “change after” date^20^. A “change after” date is defined as a dated sample lying immediately below a marked sedimentary discontinuity identified by a grain-size reversal in a floodplain fining-up sedimentary sequence, or where a peat or soil is overlain by a minerogenic sediment unit^20^. These types of stratigraphic breaks have been shown to indicate either a significant single flood event or a general increase in flood frequency and/or magnitude resulting in a change in sedimentation style. A ^14^C age classified as a “change after” date gives a *terminus post quem* for the shift in flooding regime that produced the lithofacies change. Radiocarbon dates from archaeological sites on floodplains were not considered in the analysis, in order to avoid introducing bias towards periods relating to floodplain settlement and encroachment^3^.

The ^14^C dates were calibrated using the IntCal 13 calibration curve^26^, ^14^C and OSL ages were summed using OxCal v. 4.2^27^. The combined analysis of ^14^C and OSL requires some caution in the accurate conversion to a common time scale that is particularly important where OSL ages have small errors (e.g. < ±20 years), which occurs most frequently for samples dated to recent centuries. The data are presented as a cumulative probability density function (CPDF) plot of fluvial units that can be related to periods of flooding or stability, depending on the sub-set analysed. This approach enables consistent graphical comparison of multiple individual records, as well as an effective tool to identify periods with a greater number of extreme events. The analysis of flood episodes was carried out on a regional basis as well as within two aggregated data-sets comprising the Mediterranean and northern temperate Europe (UK, central Europe and Russian plain). To objectively compare the timing of flood periods in these two aggregated data-sets, the moving average was calculated at 500 year intervals in order to reduce short-term temporal variations (Fig. 3e).

In the Mediterranean sector eight multi-centennial periods of flooding that affected at least half of the considered regions were recorded (Fig. 2c). In northwest and northern Europe (Fig. 2b), thirteen flood episodes overlapping two or more regions were identified which are more evenly distributed over time (Fig. 2b). In the entire geographical domain, nine flood episodes (registered in six or more regions) were identified (Figs 2a and 3c).

## Additional Information

**How to cite this article**: Benito, G. *et al*. Recurring flood distribution patterns related to short-term Holocene climatic variability. *Sci. Rep.* **5**, 16398; doi: 10.1038/srep16398 (2015).

## Supplementary Material

Supplementary Information

## Figures and Tables

**Figure 1:**
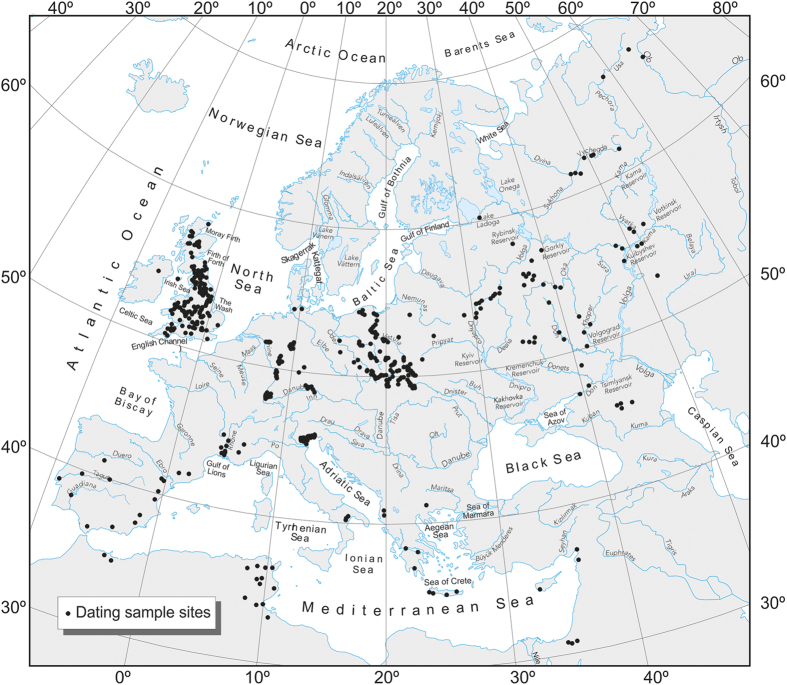
Location of sites in Europe and North Africa with ^14^C and OSL dated river flood sediments. Site locations from HEX project database processed in ArcMap10.

## References

[b1] Ely L. L., Enzel Y., Baker V. R. & Cayan D. R. A 5000-year record of extreme floods and climate change in the southwestern United States. Science 262, 410–412 (1993). 17789949 10.1126/science.262.5132.410

[b2] Macklin M. G. . Past hydrological events reflected in the Holocene fluvial record of Europe. Catena 66, 145–154 (2006).

[b3] Macklin M. G. & Lewin J. River sediments, great floods and centennial-scale Holocene climate change. J. Quat. Sci. 18, 101–105 (2003).

[b4] De Lange G. J. . Synchronous basin-wide formation and redox-controlled preservation of a Mediterranean sapropel. Nat. Geosci. 1, 606–610 (2008).

[b5] Lauterbach S. . A sedimentary record of Holocene surface runoff events and earthquake activity from Lake Iseo (Southern Alps, Italy). Holocene 22, 749–760 (2012).

[b6] Magny M. . North-south palaeohydrological contrasts in the central Mediterranean during the Holocene: tentative synthesis and working hypotheses. Clim. Past 9, 2043–2071, 10.5194/cp-9-2043-2013 (2013).

[b7] Clegg B. F., Kelly R., Clarke G. H., Walker I. R. & Hu F. S. Nonlinear response of summer temperature to Holocene insolation forcing in Alaska. Proc. Natl. Acad. Sci. 108, 19299–19304, 10.1073/pnas.1110913108 (2011). 22084085 PMC3228435

[b8] Bond G. . Persistent Solar Influence on North Atlantic Climate During the Holocene. Science 294, 2130–2136 (2001). 11739949 10.1126/science.1065680

[b9] Lamb H. H. Climate, present, past and future. (Methuen and Co., 1972).

[b10] Mayewski P. A. & Maasch K. A. Recent warming inconsistent with natural association between temperature and atmospheric circulation over the last 2000 years. Clim. Past Discuss. 2, 327–355, 10.5194/cpd-2-327-2006 (2006).

[b11] Benito G., Macklin M. G., Zielhofer C., Jones A. F. & Machado M. J. Holocene flooding and climate change in the Mediterranean. Catena 130, 13–33 (2015).

[b12] Roberts N. . Palaeolimnological evidence for an east–west climate see-saw in the Mediterranean since AD 900. Glob. Planet. Change 84–85, 23–34 (2012).

[b13] Rimbu N., Lohmann G., Kim J. H., Arz H. W. & Schneider R. Arctic/North Atlantic Oscillation signature in Holocene sea surface temperature trends as obtained from alkenone data. Geophys. Res. Lett. 30, 10.1029/2002GL016570 (2003).

[b14] O’Brien S. R. . Complexity of Holocene Climate as Reconstructed from a Greenland Ice Core. Science 270, 1962–1964 (1995).

[b15] Woollings T., Lockwood M., Masato G., Bell C. & Gray L. Enhanced signature of solar variability in Eurasian winter climate. Geophys. Res. Lett. 37, 10.1029/2010GL044601 (2010).

[b16] Martin-Puertas C. . Regional atmospheric circulation shifts induced by a grand solar minimum. Nat. Geosci. 5, 397–401 (2012).

[b17] Rossato S., Fontana A. & Mozzi P. Meta-analysis of a Holocene 14C database for the detection of paleohydrological crisis in the Venetian–Friulian Plain (NE Italy). Catena 130, 34–45 (2015).

[b18] Panin A. & Matlakhova E. Fluvial chronology in the East European Plain over the last 20 ka and its palaeohydrological implications. Catena 130, 46–61 (2015).

[b19] Johnstone E., Macklin M. G. & Lewin J. The development and application of a database of radiocarbon-dated Holocene fluvial deposits in Great Britain. Catena 66, 14–23 (2006).

[b20] Macklin M. G., Jones A. F. & Lewin J. River response to rapid Holocene environmental change: evidence and explanation in British catchments. Quat. Sci. Rev. 29, 1555–1576 (2010).

[b21] Starkel L., Soja R. & Michczyńska D. J. Past hydrological events reflected in Holocene history of Polish rivers. Catena 66, 24–33 (2006).

[b22] Thorndycraft V. R. & Benito G. The Holocene fluvial chronology of Spain: evidence from a newly compiled radiocarbon database. Quat. Sci. Rev. 25, 223–234 (2006).

[b23] Piccarreta M., Caldara M., Capolongo D. & Boenzi F. Holocene geomorphic activity related to climatic change and human impact in Basilicata, Southern Italy. Geomorphology 128, 137–147 (2011).

[b24] Zielhofer C. & Faust D. Mid- and Late Holocene fluvial chronology of Tunisia. Quat. Sci. Rev. 27, 580–588 (2008).

[b25] Hoffmann T., Lang A. & Dikau R. Holocene river activity: analysing 14C-dated fluvial and colluvial sediments from Germany. Quat. Sci. Rev. 27, 2031–2040 (2008).

[b26] Reimer P. J. . IntCal13 and Marine13 Radiocarbon Age Calibration Curves 0–50,000 Years cal BP. Radiocarbon 55, 1869–1887 (2013).

[b27] Bronk Ramsey C. Bayesian Analysis of Radiocarbon Dates. Radiocarbon 51, 337–360 (2009).

[b28] Berger A. & Loutre M. F. Insolation values for the climate of the last 10 million years. Quat. Sci. Rev. 10, 297–317 (1991).

[b29] Mayewski P. A. . Major features and forcing of high-latitude northern hemisphere atmospheric circulation using a 110,000-year-long glaciochemical series. J. Geophys. Res.: Oceans 102, 26345–26366 (1997).

[b30] Steinhilber F., Beer J. & Fröhlich C. Total solar irradiance during the Holocene. Geophys. Res. Lett. 36, 10.1029/2009GL040142 (2009).

[b31] Usoskin I. G., Solanki S. K. & Kovaltsov G. A. Grand minima and maxima of solar activity: new observational constraints. Astron. Astrophys. 471, 301–309 (2007)

