# Supplementary material for: Recurring flood distribution patterns related to short-term Holocene climatic variability
Source: Sci Rep. 2015 Nov 9;5:16398. doi: 10.1038/srep16398 (PMC4637870; doi:10.1038/srep16398)
Supplement: Supplementary Information [file srep16398-s1.pdf]

Supplemental Material for the manuscript:

Recurring flood distribution patterns related to short-term Holocene climatic variability

Gerardo Benito[1*], Mark G. Macklin[2,3], Andrei Panin[4], Sandro Rossato[5], Alessandro Fontana[5], Anna F. Jones[6], Maria J. Machado[1], Ekaterina Matlakhova[4], Paolo Mozzi[5], and Christoph Zielhofer[7]

[1] Museo Nacional de Ciencias Naturales, CSIC, Serrano 115 bis, 28006 Madrid, Spain.
[2] Centre for Catchment and Coastal Research and the River Basin Dynamics and Hydrology Research Group, Department of Geography and Earth Sciences, Aberystwyth University, Ceredigion SY23 3DB, UK.
[3] Innovative River Solutions, Institute of Agriculture and Environment, Massey University, Private Bag 11 222, Palmerston North, 4442, New Zealand
[4] Lomonosov Moscow State University, Faculty of Geography, Lengory 1, Moscow, 119991, Russia.
[5] University of Padua, Department of Geosciences, Via Gradenigo, 6. 35131 Padova, Italy.
[6] School of Geography, Planning and Environmental Policy, University College Dublin, Newman Building, Belfield, Dublin 4, Ireland.
[7] Leipzig University, Institute of Geography, Johannisallee 19a, 04103 Leipzig, Germany.

* Correspondence:
Prof. Gerardo Benito,
Museo Nacional de Ciencias Naturales,
Spanish Research Council-CSIC,
Serrano 115 bis,
28006 Madrid, Spain

benito@mncn.csic.es
+34 917822083

General flood episodes	United Kingdom[20] cal. yr BP n = 252	Germany[25] cal. yr BP n = 401	Poland[21] cal. yr BP n =331	East European Plain[18] (Russia) cal. yr BP n = 206	Eastern Mediterranean cal. yr BP n =78	Tunisia[24] cal. yr BP n =103	NE Morocco cal. yr BP n =30	Southern Italy[23] cal. yr BP n =34	N-Eastern Italy[17] cal. yr BP n =136	Southern France cal. yr BP n =44	W-Iberian Peninsula[11] cal. yr BP n =86	E-Iberian Peninsula[11] cal. yr BP n =37
300-100	300-0			250-100	260-85	300-200		200-100		250-100	500-150	600-150
										600-400		
700-500	800-500			900-500	900-600	700-500						
1000-800	900-800<br>1100-1000	1075-0	1000-800			900-800		1050-800	1100-800	950-800	1100-800	1050-800
					1200-1100				1300-1100			
1500-1400	1500-1100		1400-1300			1600-1300		1500-1400	1600-1400	1500-1350		
			1900-1800		1900-1600			1700-1600	1900-1700			
2400-2100	2300-2000	2275		2300-1900			2500-2100	2350-1850	2300-2100	2300-1750	2800-2250	2450-2150
2900-2800	2900-2800	2770	2900-2800	3000-2900		2850-2350						2850-2700
3400-3100		3300		3400-3000	3400-3200	3300-3000	3300-3100	3450-3000				
	3600-3400											
					4100-3700	4100-3700	4200-3700					
	4300-4200	4200						4200-4100				
				4500-4400								
4800-4500	4900-4500		4800-4700			4800-4500		4800-4600	4700-4500	4800-4500	4800-4500	4850-4700
	5300-5100			5250-4900								
									5500-5300			
	6000-5700	5640	5800-5700									
	6300-6200			6200-6000		6200-6000			6900-6200			
	6900-6500							7400-6760				
7600-7300	7600-7300	7475		7900-7200	7800-7150	7600-7150			7500-7200	7800-7150		
	7800-7700		7700-7600									
		8250-8000	8400						8400-8300			
		9000-8500		9100-8600			8900-8700					
	9400-9100		9600-9500								9500-9200	
	10700-10400			10700-10500								

Table S1. Centennial and multi-centennial episodes of flooding in Europe and the Mediterranean regions based on analysis of ^{14}C and OSL-dated Holocene fluvial units.

Note: overlapping episodes (at least 6 regions involved) are highlighted. cal.—calibrated.

Region	Proximity Matrix (Jaccard's Measure)											
	United Kingdom	Germany	Poland	East European Plain	Eastern Mediterranean	Tunisia	NE Morocco	Southern Italy	N-Eastern Italy	Southern France	W-Iberian Peninsula	E-Iberian Peninsula
United Kingdom	1.000	**.357**	.160	.281	.188	.278	.063	.273	**.333**	**.464**	.194	.267
Germany	**.357**	1.000	.105	**.320**	.250	.152	.040	.172	.167	.269	.261	**.364**
Poland	.160	.105	1.000	.043	.105	.160	.000	.190	.188	**.333**	.250	.235
East European Plain	.281	**.320**	.043	1.000	.269	.206	.160	**.370**	.107	**.333**	.032	.138
Eastern Mediterranean	.188	.250	.105	.269	1.000	.267	.238	.214	.167	.138	.036	.034
Tunisia	.278	.152	.160	.206	.267	1.000	**.308**	.235	.161	.281	**.370**	.267
NE Morocco	.063	.040	.000	.160	.238	**.308**	1.000	.250	.091	.074	.087	.182
Southern Italy	.273	.172	.190	**.370**	.214	.235	.250	1.000	.280	**.423**	.179	.214
N-Eastern Italy	**.333**	.167	.188	.107	.167	.161	.091	.280	1.000	**.348**	.227	.217
Southern France	**.464**	.269	**.333**	**.333**	.138	.281	.074	**.423**	**.348**	1.000	.280	**.320**
W-Iberian Peninsula	.194	.261	.250	.032	.036	**.370**	.087	.179	.227	.280	1.000	**.611**
E-Iberian Peninsula	.267	**.364**	.235	.138	.034	.267	.182	.214	.217	**.320**	**.611**	1.000

Table S2. Proximity matrix based on Jaccard's similarity coefficient. The highest similarity value equals 1. The variable data sets include the last 5000-yr record for the twelve regional datasets with binary information of temporal flood activity in 100-yr bins. Classes: 1: Flood activity 100-yr bins; 0: Non-activity 100-yr bins. Percentage of class 1 values in datasets. UK 45%, Germany 30%, Poland 11.8%, East European Plain 35.5%, Eastern Mediterranean 29.4%, Tunisia 45.1%, NE Morocco 27.5%, Southern Italy 35.3%, North-eastern Italy 25.5%, Southern France 37.3%; W Iberia 25.5%; E Iberia 27.5%. Correlation values larger than 0.3 are printed in bold.
